# Scanning electron microscopy of polycystic liver disease

**DOI:** 10.1007/s10157-018-1550-x

**Published:** 2018-03-08

**Authors:** Kazutaka Kojima, Masaji Hashimoto, Yoshifumi Ubara

**Affiliations:** 1 Department of Gastrointestinal Surgery, Toranomon Hospital, Tokyo, Japan; 2 Nephrology Center, Toranomon Hospital Kajigaya, 1-3-1, Kajigaya, Takatsu, Kawasaki, 213-8587 Kanagawa Japan; 3 Okinaka Memorial Institute for Medical Research, Toranomon Hospital, Tokyo, Japan

**Keywords:** Polycystic liver disease (PCLD), ADPKD (autosomal dominant polycystic kidney disease), Fenestration surgery, Scanning electron microscopy

## Abstract

Resected specimens of PCLD by laparoscopic fenestration surgery were evaluated by scanning electron microscopy. Epithelium lining the largest cyst (26 cm in size) showed prominent villous proliferation with positivity of Ki-67, while the epithelium of the small cyst (3 cm in size) showed slight proliferation (smooth) with small positivity of Ki-67.

A 69-year-old woman with ADPKD was admitted to our hospital for evaluation of abdominal distention due to PCLD. Resected specimens by laparoscopic fenestration surgery were evaluated by scanning electron microscopy. Epithelium lining the largest cyst (26 cm in size) (Fig. 1a) showed prominent villous proliferation with positivity of Ki-67 (Fig. 2a), while the epithelium of the small cyst (3 cm in size) (Fig. 1b) showed slight proliferation (smooth) with small positivity of Ki-67 (Fig. 2b). We believe that villous change of the cyst epithelium may be closely associated with largest cyst volume via the proliferation of cyst lining cells in PCLD.

**Fig. 1:**
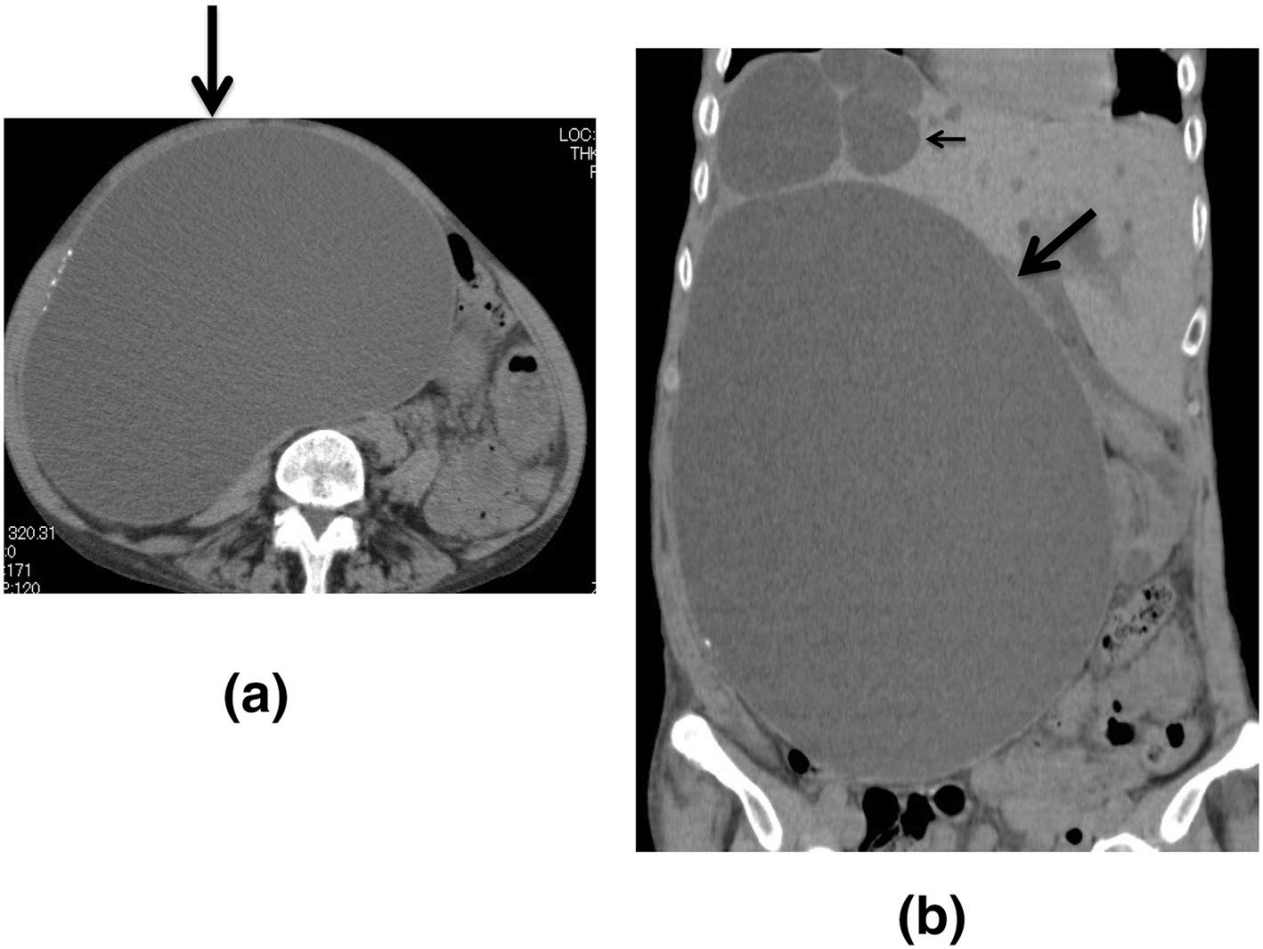
Computed tomography showed a huge cyst, and contracted kidneys with multiple small cysts. Laparoscopic fenestration surgery was performed for huge cyst measuring 26.1 cm × 23.7 cm × 16.1 cm (large arrow) and small cyst measuring 3.1 cm × 2.9 cm × 2.6 cm (small arrow). **a** Axial section, **b** coronal section

**Fig. 2:**
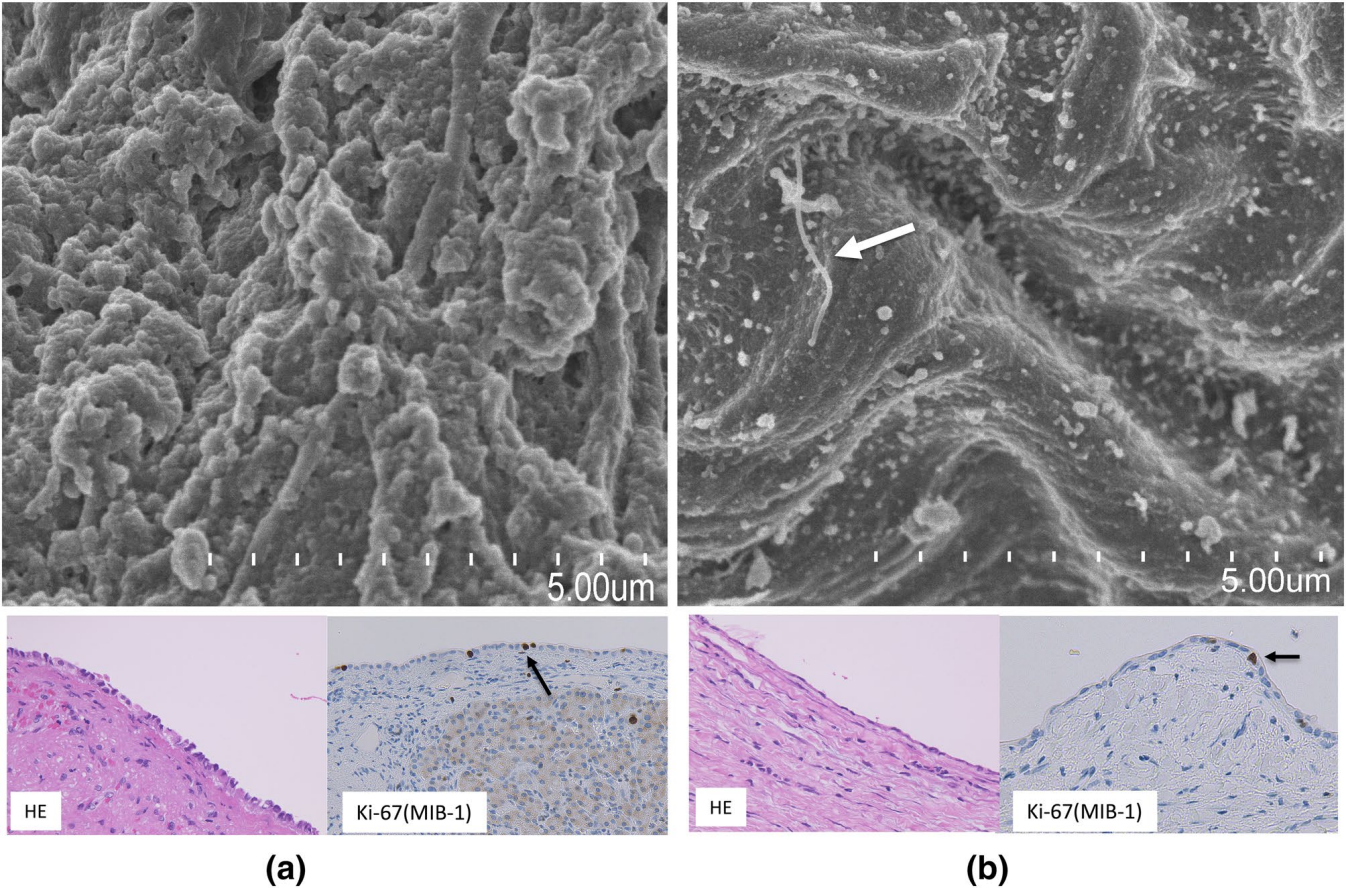
Scanning electron microscopy of the largest cyst **a** and small cyst, **b** shows prominent villous proliferation (without cilium) (**a**) and slight proliferation (with cilium) (large arrow) (**b**), respectively. Inset shows hematoxylin and eosin stain (HE) and Ki-67 (MIB-1) of cyst wall; **a** the largest cyst shows high length epithelium with positivity of Ki-67 (small arrows), and **b** small cyst shows flat epithelium with small positivity of Ki-67 (small arrow)

